# Decellularized dermis extracellular matrix alloderm mechanically strengthens biological engineered tunica adventitia-based blood vessels

**DOI:** 10.1038/s41598-021-91005-9

**Published:** 2021-05-31

**Authors:** Bijal Patel, Bryan T. Wonski, Dan M. Saliganan, Ali Rteil, Loay S. Kabbani, Mai T. Lam

**Affiliations:** 1 Department of Biomedical Engineering, Wayne State University, 818 W. Hancock Street, Detroit, MI 48201 USA; 2 Department of Vascular Surgery, Henry Ford Health System, 2799 W Grand Blvd, Detroit, MI 48202 USA

**Keywords:** Biomedical engineering, Translational research, Biologics, Regenerative medicine, Tissue engineering

## Abstract

The ideal engineered vascular graft would utilize human-derived materials to minimize foreign body response and tissue rejection. Current biological engineered blood vessels (BEBVs) inherently lack the structure required for implantation. We hypothesized that an ECM material would provide the structure needed. Skin dermis ECM is commonly used in reconstructive surgeries, is commercially available and FDA-approved. We evaluated the commercially-available decellularized skin dermis ECM Alloderm for efficacy in providing structure to BEBVs. Alloderm was incorporated into our lab’s unique protocol for generating BEBVs, using fibroblasts to establish the adventitia. To assess structure, tissue mechanics were analyzed. Standard BEBVs without Alloderm exhibited a tensile strength of 67.9 ± 9.78 kPa, whereas Alloderm integrated BEBVs showed a significant increase in strength to 1500 ± 334 kPa. In comparison, native vessel strength is 1430 ± 604 kPa. Burst pressure reached 51.3 ± 2.19 mmHg. Total collagen and fiber maturity were significantly increased due to the presence of the Alloderm material. Vessels cultured for 4 weeks maintained mechanical and structural integrity. Low probability of thrombogenicity was confirmed with a negative platelet adhesion test. Vessels were able to be endothelialized. These results demonstrate the success of Alloderm to provide structure to BEBVs in an effective way.

## Introduction

Current technologies in vascular tissue engineering rely on polymer tubes as a scaffolding material or cell sheets wrapped into a tube. Leading approaches share the same basic principles- seeding of vascular smooth muscle cells onto a tubular scaffold such as a polymer^1–3^ or hydrogel^4–6^, followed by weeks of mechanical conditioning for extracellular matrix deposition to increase vessel strength, and finally decellularization to remove immunogenic components. However, polymers can lead to graft failure due to the body’s natural foreign body response leading to a persistent inflammatory response^7^. The hydrogel grafts are completely biological and thus illicit minimal immune response, however presently the most effective current way to achieve sufficient strength is to subject these vessels to mechanical conditioning for several weeks to months, which has impeded manufacturability and translatability. Another common approach to engineer blood vessels is to roll cell sheets into tubes creating a completely biological construct^7–8^. However, this approach still requires weeks of strength conditioning. A more efficient approach for introducing the strength is needed for implantation.

We hypothesized that using a human-derived, biological material for mechanical strength as opposed to polymers commonly used in the field will provide an effective support structure that will meet the mechanical needs of a vascular graft and minimize adverse immune response. In a native blood vessel, the adventitia outer layer is key to providing structural integrity^9–11^. The extracellular matrix of the adventitia allows for a vessel to withstand high pressures preventing vessel rupture^12^. The strength derives from the significant type I collagen content, which is a load bearing extracellular matrix protein that is able to resist high pressure forces^13–16^. The adventitia also contains elastin to aid in elasticity and vessel distension^17^. Collagen is found in many organs, with especially high levels in the skin. Specifically, the ECM of the skin dermis consists of type I collagen and elastin to aid in strength and elasticity^18^, thus providing an ECM similar to that of the adventitia. Decellularized dermis ECM has been used for years in reconstructive surgeries and for wound care, with several products currently on the market^19^. One product in particular, Alloderm, has been used extensively in the clinic with much success. Alloderm is a human allograft, and has been used for reconstructive surgeries, complex abdominal wall hernia repair, soft-tissue defect augmentation, rhinoplasty, and vaginal repair^18–22^. Alloderm has also been investigated for other uses such as for tendon repair, stress urinary incontinence and pelvic organ prolapse repair^23–24^. The mechanical properties of the ECM of Alloderm, and the fact that it is decellularized thus reducing inflammatory and rejection risk, makes it ideal for tissue engineering application^20,25–27^. Thus, we proposed to employ the advantageous properties of Alloderm to mimic the adventitia’s role and provide strength to engineered blood vessels. This type of application of a decellularized matrix is a departure from the typical use of decellularized matrices as a scaffold for use in its tissue of origin. Our approach represents an innovative application of a decellularized ECM for use as a reinforcement material in an engineered tissue of a different tissue origin. Our objective was to mimic the natural strength component of a native blood vessel by substituting a comparable ECM.

Using our lab’s unique method for generating completely biological engineered blood vessels (BEBVs) as our base structure^28–30^, Alloderm was integrated into our protocol. In our methods, vascular cells are formed into ring structures, which are then stacked into tubular form to form the vessel. This method is termed the Ring Stacking Method (RSM). This method is highly modifiable and allows for the integration of reinforcing materials such as Alloderm. Once the Alloderm material was successfully added to our RSM protocol, the resultant engineered rings and vessel structures were tested mechanically. Tensile tests revealed that the Alloderm-integrated engineered vessels reached a tensile strength of 1500 ± 334 kPa, which is in the range of a native vessel’s tensile strength of 1430 ± 604 kPa^12^. Burst pressure reached 80 mmHg, indicating that the decellularized ECM was able to increase the completely biological engineered vessels blood pressure capabilities to within the diastolic blood pressure range. Histological analysis showed that the overall collagen amount and degree of fiber maturity were significantly increased, elements that both contribute to tissue strength. These results demonstrate the success of Alloderm to provide sufficient mechanical support to the BEBVs in a much more efficient and effective way than current approaches in the field. The novelty of this approach is in achievement of clinical requirements for engineered vessel mechanical strength using decellularized ECM, progressing this type of technology that much closer to patient use.

## Methods

### Cell culture

Human dermal fibroblasts (HuDFs) (PCS-201-012, ATCC, Manassas, VA) were used to make adventitia-based engineered vessels. Dermal fibroblasts were chosen in foresight of patient application of autologous cells. Passages up to 15 were able to form consistent rings/vessels. Fibroblast growth media (GM) consisting of 89% Dulbecco’s Modified Eagle Medium (DMEM), 10% fetal bovine serum, and 1% antibiotic/antimycotic was used to expand and maintain the cell culture. Cells were trypsinized at roughly 90% confluency and used to make the rings and vessel structures.

Human umbilical vein endothelial cells (HUVECs) (PCS-100-013, ATCC) were used to form the intima in the Alloderm vessels. HUVECs were cultured in HUVEC growth media consisting of 93%—131 media; 5% FBS and l-Glutamine (200 mM); 1% antibiotic/antimycotic; 50 µg/mL ascorbic acid; 1 µg/mL hydrocortisone; 4 µg/mL heparin sulfate; 15 ng/mL insulin-like growth factor-1 (IGF-1); and 5 ng/mL of vascular endothelial growth factor (VEGF), epidermal growth factor (EGF), and fibroblast growth factor-basic (FGF-b).

### Human saphenous veins

Human saphenous veins were obtained from diabetic patients undergoing limb amputation between years 2018–2020. The study was approved by the Human Institutional Review Boards of both entities of Wayne State University and Henry Ford Health System (IRBs 054514M1E and 10744, respectively). Informed consent was obtained from all 6 patients who donated tissues. The tissues were obtained in accordance with relevant guidelines and regulations.

### Human femoral artery

Fully intact human femoral arteries were harvested from fresh, non-treated cadavers donated to the Wayne State University Body Bequest Program. Arteries were tensile tested to determine mechanical properties and histologically processed to assess tissue morphology.

### Assembly of ring formation plates

A 60 mm petri dish was used as the basis for forming the rings. Poly(dimethylsiloxane) (PDMS) polymer (1064291, Dow Corning, Midland, MI) was used to coat plates at 3 mL per plate. Posts were made and adhered to the plates centrally to provide a structure around which the rings would form. The posts were punched from PDMS bulk material using 6 mm diameter biopsy punches to fabricate 6 mm lumen vessels. Base plates were coated with PDMS, followed by placement of the post centrally in the plate and oven cured at 60 °C for 1 h. Plates were sterilized with a 30 min 70% ethanol soak followed by 30 min of UV sterilization under the bio-hood.

### Hydrogel formulation

A provisional hydrogel was used to secure the cell sheet as it aggregated around the central post to form the ring structures. Hydrogels rapidly degrade in 2–4 weeks hence serving as a temporary support. Fibrin gel was identified as an ideal option as it is naturally found in the body. Fibrin hydrogels were formed using thrombin, fibrinogen, and hydrogel media^28^. A 4:1 ratio of 20 mg/mL bovine fibrinogen (151122, MP Biomedicals LLC, OH) to 100 U/mL bovine plasma thrombin (7592, BioVision, Milpitas, CA) was prepared. Hydrogel media consisted of 88.8% DMEM, 0.1% TGF-β and ascorbic acid, 10% fetal bovine serum, and 1% antibiotic/antimycotic.

### Alloderm preparation

Alloderm was obtained from LifeCell Corporation (Allergan, CA). Biopsy punches were used to cut 6 mm holes into Alloderm. A scalpel was used to cut a 3–3.5 mm width around the 6 mm hole. The final dimensions of the donut shaped Alloderm was an inner diameter of 6 mm and outer diameter around 12–13 mm. Alloderm donuts were sterilized in a bio-hood using UV sterilization for 15 min on both sides and kept sterile until ready to use.

### Ring formation

Cultured fibroblasts were trypsinized from pates and re-suspended in growth media. Each prepared PDMS ring plate was seeded with 1.5 × 10^6^ fibroblast cells in 4 mL GM, 150 µg ascorbic acid and 0.01 ng TGF-β either onto fibrin gel directly (basic rings/vessels) or fibrin gel topped with Alloderm (ECM-integrated rings/vessels). Media was changed one day after seeding, and subsequently every 3 days for 14 days. Each media change was supplemented with fresh ascorbic acid and TGF-β.

### Fabrication of dermis-integrated engineered vessels

To create the vascular grafts, tubular structures were created by stacking 3 or more engineered rings. Alloderm adventitia vessels were created using our lab’s Ring Stacking Method (RSM). In the RSM, engineered vascular rings are stacked around a 1.2 cm long 3D printed post placed centrally in custom made 8 cm tall plates. Engineered vessels made of stacks of 3 rings were used for histology and stacks of 6 rings were used for mechanical testing. Rings were temporarily adhered to one another using additional fibrin glue to secure the rings to each other as the cells deposited their own extracellular matrix to secure the overall vascular structure. A 1:1 volumetric ratio of 30 µL of 100 u/mL thrombin to 30 µL of 20 mg/mL fibrinogen was used for the fibrin glue. Ring stacks, i.e. vessels, were maintained in HuDF growth media supplemented with ascorbic acid and TGF-β for one week with a media change 3 days after stacking.

### Assembly of a long engineered adventitia vessel

To demonstrate the ability to scale the ring stacking method towards engineering vessels of physiologically relevant sizes, a 5 cm long adventitia vessel was created. Custom ring and vessel culture dishes were developed to accommodate the increased dimensions. The custom vessel culture dish consisted of a 50 mL conical equipped with a 5 mm diameter 3D printed polylactic acid (PLA) post holder attached to the center of the lid by PDMS. A 10 cm long, 5 mm diameter PLA post was printed, filed for smoothness, and thinly coated with PDMS to reduce ring attachment and deformation during stacking and vessel removal. A 60 mm culture plate with a central PLA post holder was utilized for stacking rings to maintain sterility of the vessel culture dish during the stacking process. All components were sterilized with 70% ethanol followed by UV radiation prior to cell culture use. Rings were formed per the Ring Formation protocol using human dermal fibroblasts. Following six days of self-organization, adventitia rings were gently removed from the wells with fine-tip forceps and transferred to the 10 cm PLA post fitted in the 60 mm stacking dish post holder. After the rings were stacked and fit closely together, 80 µL of thrombin (100 U/mL) and 80 µL of fibrinogen (20 mg/mL) were applied to the outer surface of the vessel to aid in ring-to-ring adhesion. Following ring stacking, the vessel post was transferred from the stacking dish and placed in the custom 50 mL conical post holder for culture. Forty-seven rings were used to build the 5 cm long vessel.

### Endothelialization of adventitia vessels

In preparation of endothelial cell seeding, adventitia vessels were transferred into an 8 mm diameter PDMS cylindrical shell with two removable 3D printed PLA tube fittings. Adventitia vessels were transferred from culture posts into the cylindrical shell with one fitting blocking the outlet. Human umbilical endothelial cells (HUVECs) at a concentration of 4 × 10^6^ cells/mL in HUVEC media were used to create a cell suspension. Luminal seeding was achieved by pipetting 600 µL of the endothelial cell suspension into the adventitia vessel lumen followed by sealing the PDMS shell with the second tube fitting. The cylindrical culture chamber was placed horizontally in a culture dish and incubated for 1 h. Following this period, the media with HUVECs was carefully removed by pipetting, the vessel was rotated 120°, and the endothelial seeding process was repeated until a full 360° rotation was achieved. Endothelial cell-seeded vessels were removed from the PDMS shell, placed into a traditional culture dish, and incubated in 50% HUVEC media and 50% fibroblast growth media for 24 h prior to analysis. The HUVEC-seeded Alloderm vessels were fixed in formalin for 24 h, dehydrated, embedded in paraffin, and sectioned. The vessels were stained with FITC-conjugated Ulex Europaeus (Gorse) Agglutinin I (UEA I) to mark the endothelial cell glycoproteins and glycolipids, and DAPI to mark nuclei.

### Long-term mechanics

To test long-term mechanics of the Alloderm-integrated engineered vascular tissue, rings with Alloderm (n = 10) were cultured for 4 weeks then tensile tested. Controls consisted of Alloderm rings tested immediately after formation, denoted as timepoint day 0. Rings were maintained in culture in fibroblast growth media supplemented with ascorbic acid and TGF-β. Media was changed every 3 days. Tensile testing was performed on a Mark-10 ESM 301 (Mark-10, Copiague, NY) with a 1000 N load cell at a strain rate of 0.4 mm/min until failure. Samples were formalin fixed for 24 h, dehydrated, paraffin embedded and sectioned for histological analysis.

### Mechanical testing

Tensile testing was performed using an UStretch system with a 5 N load cell (CellScale, Waterloo, Ontario, Canada) and an Instron 5943 with a 50 N and a 500 N load cell (Instron, Norwood, MA). The smaller load UStretch was used for tensile testing in the range of the original rings and vessels without Alloderm. The higher load Instron was used to tensile test the Alloderm by itself, and Alloderm-integrated rings and vessels. Rings were tensile tested 14 days after cell seeding. Two modes of tensile testing were conducted to analyze both circumferential and longitudinal vessel mechanics. Circumferential tensile testing was used to measure the hoop strength of the rings and vessels. Longitudinal testing was used to measure the strength along the length of the vessels. Samples were stretched to failure and mechanical properties of elastic modulus, ultimate tensile strength and failure strength were obtained from resultant stress–strain curves.

Circumferential tensile testing was conducted at a strain rate of 0.4 mm/min until failure. Rings with Alloderm (n = 5) and without dermis (n = 5) were tested to determine the effect of the addition of Alloderm. Alloderm donuts alone (n = 6) were tensile tested to determine their independent mechanical properties. For tensile testing, engineered vessels were built with 6 rings each. Vessels with (n = 5) and without (n = 5) Alloderm were tensile tested after a 7-day culture period following ring stacking. For control data, a human saphenous vein (cut into n = 6) obtained from a diabetic patient following amputation and a human femoral artery (cut into n = 8) obtained from un-embalmed cadaver were tensile tested.

Longitudinal tensile testing was performed at a strain rate of 0.4 mm/min until failure. Engineered vessels with (n = 5) and without Alloderm (n = 5) were tensile tested following a 7 day culture period after ring stacking. In order to attach the vessels longitudinally into the tensile tester, VetBond tissue adhesive (3 M, St. Paul, MN) was used to fix the two ends of the vessels onto sandpaper, which was then folded and placed on the Ustretch system hooks.

### Histology

Engineered rings; engineered vessels; and controls of human saphenous vein and cadaver femoral artery were histologically analyzed to determine tissue structure, ECM content and ECM organization. Tissues were processed in paraffin. Individual rings and 3-ring vessels with and without Alloderm were fixed in formalin for 48 h. Samples were stored in 70% ethanol in 5 °C until dehydrated. Samples were dehydrated in graduations of 70% to 100% ethanol over 12 h and then embedded into paraffin blocks. Hematoxylin and eosin (H&E), Masson’s Trichrome, and Picrosirius red stains were conducted on all ring groups and Alloderm donuts. H&E revealed cellular and extracellular matrix organization. DAPI stain was used to determine cellularity. Masson’s Trichrome and Picrosirius red stains showed collagen organization and total content in the rings and Alloderm donuts. Collagen quantification for red and blue stained collagen from picrosirius red and trichome, respectively, were quantified as a percentage of total cross-sectional area using ImageJ.

### Polarized light

Picrosirius red stained samples of rings and Alloderm donuts were observed under polarized light to determine collagen maturity of each sample. Birefringence images were captured using an Axiovert 200 microscope (Carl Zeiss, Oberkochen, Germany). Mature, thicker collagen fibers appear orange and red under polarized light. Immature, thinner collagen fibers appear yellow and green under polarized light. Polarized light images were quantified for percent cross-sectional area of red, yellow and green fibers using ImageJ.

### Hemodynamic testing

The hemodynamic capabilities of the engineered vessels were assessed by burst pressure testing. Burst pressure gives an indication of the maximum pressure the ECM-integrated BEBVs can withstand. Alloderm-integrated vessels were subjected to pulsatile flow with cell culture media in a custom-made bioreactor with a peristaltic pump (WT600-2J, Longer Precision Pump Cp. Ltd, Boonton, NJ), a glass media reservoir, polymer tubing, a custom-made bioreactor chamber, and 3D printed vessel holders. Vessels secured onto 3D printed vessel holders using VetBond. Alloderm vessels (n = 5) were subjected to increasing fluid pressure until failure.

### Suture retention

Suture retention tests were conducted to examine the graft’s ability to mechanically retain a suture placed using standard surgical technique. These tests were conducted using the UStretch system. Engineered vessels with (n = 5) and without Alloderm (n = 5) were tested following a 7 day culture period after ring stacking. Sutures sized 6–0 proline were sutured through the bottom end of the vessel and then fixed to the bottom hook of the UStretch system using sandpaper and gorilla glue. The top stationary hook on the Ustretch system held the engineered vessels fixed in place using VetBond and sandpaper. The suture was pulled at a strain rate of 0.4 mm/min until the failure. Force output during the duration of the test was recorded and plotted versus displacement.

### Thrombogenicity platelet adhesion assay

To test the thrombogenicity of the engineered vessels, vessels were incubated with fresh human platelets for 24 h in culture. The human platelets were donated by the Henry Ford Hospital System Blood Bank (Detroit, MI). Following the 24 h platelet culture, vessels were removed from the platelet solution, formalin fixed for 24 h, dehydrated, paraffin embedded, and sectioned. Platelet adherence to the vessel was assayed by immunostaining using CD41 platelet antibody. The CD41 antibody developed by Rockefeller University was obtained from the Developmental Studies Hybridoma Bank, created by the NICHD of the NIH and maintained at The University of Iowa, Department of Biology, Iowa City, IA 52242. Controls consisted of platelets embedded in fibrin hydrogel.

### Statistics

Statistical analyses were conducted using SPSS (IBM, Armonk, New York). Results were presented as means with standard error of means. One-way ANOVA were performed to compare material properties determined from circumferential tensile testing of rings and vessels. Tukey B post-hoc test was used to determine significance between groups. Student’s t-test was performed to compare engineered vessels with and without Alloderm for longitudinal tensile testing, suture retention and burst pressure comparisons. An independent sample t-test was used to compare material properties of the 4-week culture rings to newly formed rings. The alpha level was set to 0.05.

## Results

### Ring and vessel formation

**Figure 1:**
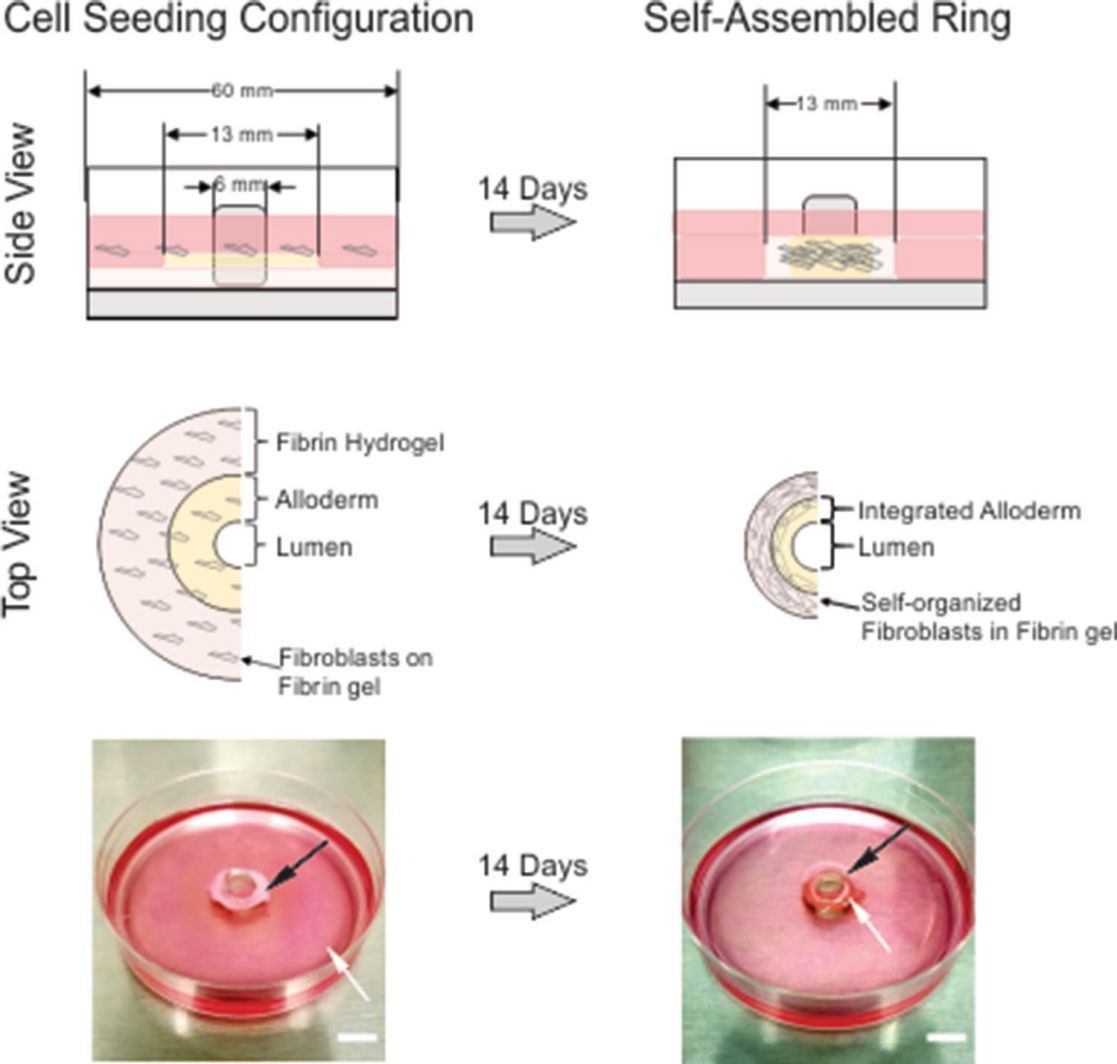
Diagram of self-assembled rings with incorporated Alloderm. Alloderm was integrated into the engineered vascular rings by placing the ECM material into the plate prior to cell seeding. Fibroblasts were seeded on top of the Alloderm and hydrogel, and the cells were able to infiltrate both the Alloderm ECM and hydrogel. Plate images show the progression of the engineered vascular ring formation 1 day and 14 days following seeding, showing the cell monolayer with hydrogel (edge indicated by white arrows) and the location of the Alloderm (black arrow) in the final ring tissue. Scale bar = 1 cm.

The Alloderm decellularized dermis was incorporated into our tissue engineered vessel protocol^28–30^ as depicted in Fig. 1. The Alloderm material exhibited some additional stiffness compared to fresh dermis tissue determined by observation, likely due to the proprietary treatment protocol for commercialization^31^. The human dermal fibroblasts were able to infiltrate the Alloderm material once seeded. Alloderm did not hinder ring formation and integrated into the lumen side of the ring structures and vessels.

### Ring mechanics

**Figure 2:**
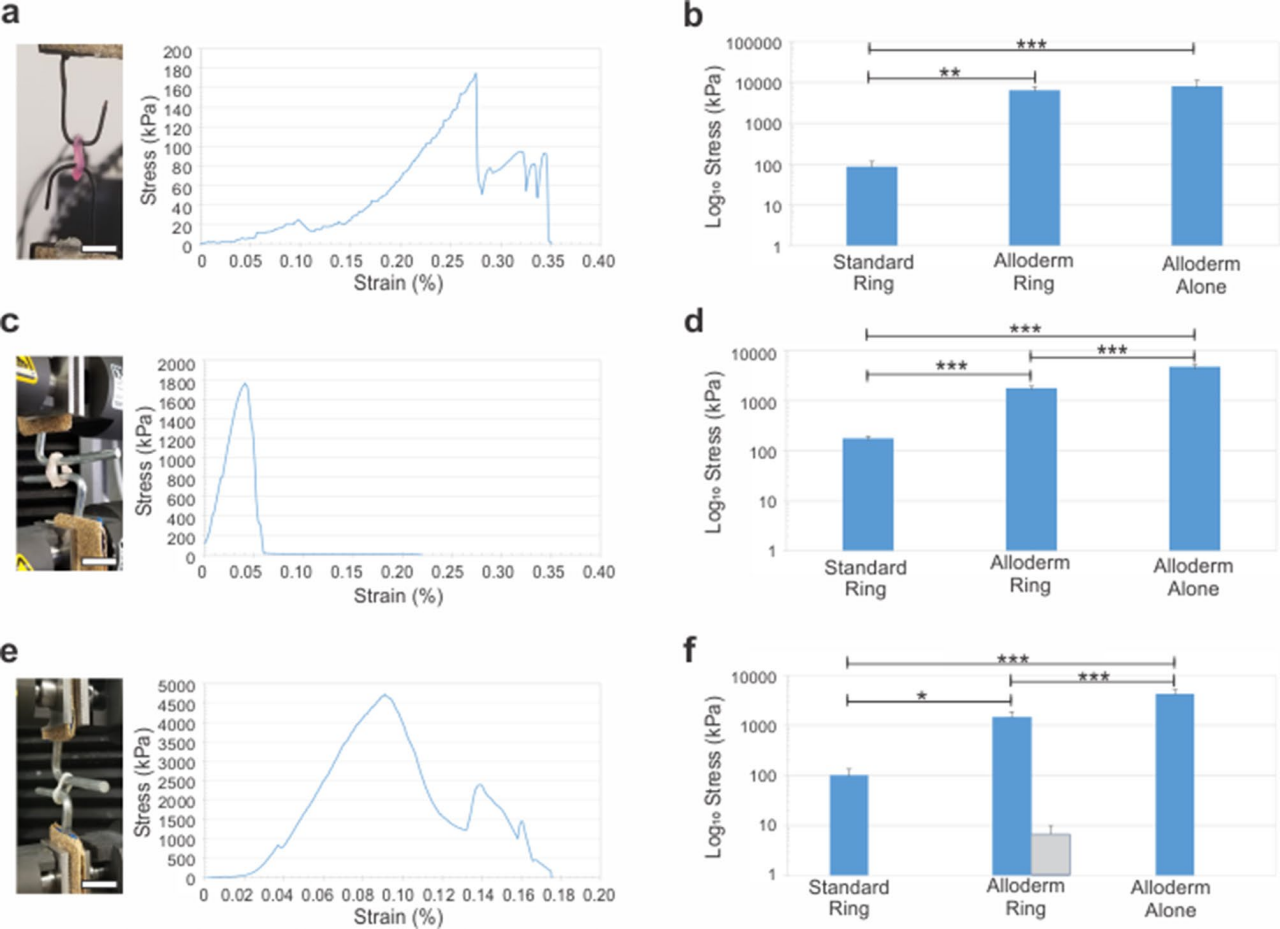
Significantly increased mechanical properties with inclusion of Alloderm into engineered vascular rings. (**a,c,e**) Average stress–strain curve of standard rings (n = 5), Alloderm rings (n = 5) and Alloderm alone (n = 5). (**b**) Elastic modulus, (**d**) ultimate tensile strength and (**f**) failure strength shown for all groups. Elastic modulus, ultimate tensile strength and failure strength significantly improved with inclusion of Alloderm into the rings**.** Two failure strengths were exhibited by the Alloderm rings (**f**), indicating first rupture of the Alloderm (blue bar) and complete tissue failure of the rest of the ring composed of the cells and hydrogel (gray bar). Compared to Alloderm alone, Alloderm rings exhibited significantly lower elastic modulus, ultimate tensile strength, and failure strength. *p < 0.01; **p < 0.001; ***p < 0.0001. Scale bars = 1 cm.

Circumferential ring tensile mechanics significantly improved with inclusion of Alloderm in the rings and vessels. Average stress–strain curves for rings without Alloderm, rings with Alloderm and Alloderm donuts are shown in Fig. 2 and material properties are summarized in Table 1. Average elastic modulus, ultimate tensile strength and failure strength for rings without Alloderm (n = 5) were 89.1 ± 27.5 kPa, 177 ± 21.4 kPa and 101 ± 34.8 kPa, respectively. Rings with Alloderm (n = 5) had an elastic modulus of 6630 ± 1510 kPa and an ultimate tensile strength of 1770 ± 221 kPa. Alloderm rings exhibited two main rupture points, consisting of the Alloderm completely tearing first at 1500 ± 372 kPa, followed by the remaining cells and hydrogel structure tearing to failure at 6.75 ± 3.25 kPa (Supplemental Video 1). Average elastic modulus, ultimate tensile strength and failure strength for Alloderm donuts alone (n = 4) were 8250 ± 3360 kPa, 4730 ± 628 kPa and 4390 ± 848 kPa, respectively. The percent elongation of rings, Alloderm rings and Alloderm donuts was 310 ± 29.8%, 162 ± 48.3% and 89.9 ± 16.3%, respectively.

**Table 1:** Average circumferential ring mechanical properties.

Group	E (kPa)	UTS (kPa)	FS primary (kPa)	FS secondary (kPa)	Percent elongation (%)
Standard Rings (n = 5)	89.1 ± 27.5^a,c^	177 ± 21.4^a,c^	101 ± 34.8^a,c^	N/A	310. ± 29.8^a,c^
Alloderm Rings (n = 5)	6630 ± 1510^a^	1770 ± 221^b,a^	1500 ± 372^b,a^	6.75 ± 3.25	162 ± 48.3^b,a^
Alloderm Alone (n = 4)	8250 ± 3360^c^	4730 ± 628^b,c^	4390 ± 848^b,c^	N/A	89.9 ± 16.3^b,c^

^a^Statistically significant difference between standard rings and Alloderm rings (E: p ≤ 0.0010; UTS: p < 0.0001; FS Primary: p < 0.01; Percent Elongation: p < 0.001).

^b^Statistically significant difference between Alloderm rings and Alloderm alone (E: not significant; UTS: p < 0.0001; FS Primary: p < 0.0001; Percent Elongation: p < 0.05).

^c^Statistically significant difference between standard rings and Alloderm alone (E: p < 0.0001; UTS: p < 0.0001; FS Primary: p < 0.0001; Percent Elongation: p < 0.001).

### Vessel mechanics

**Figure 3:**
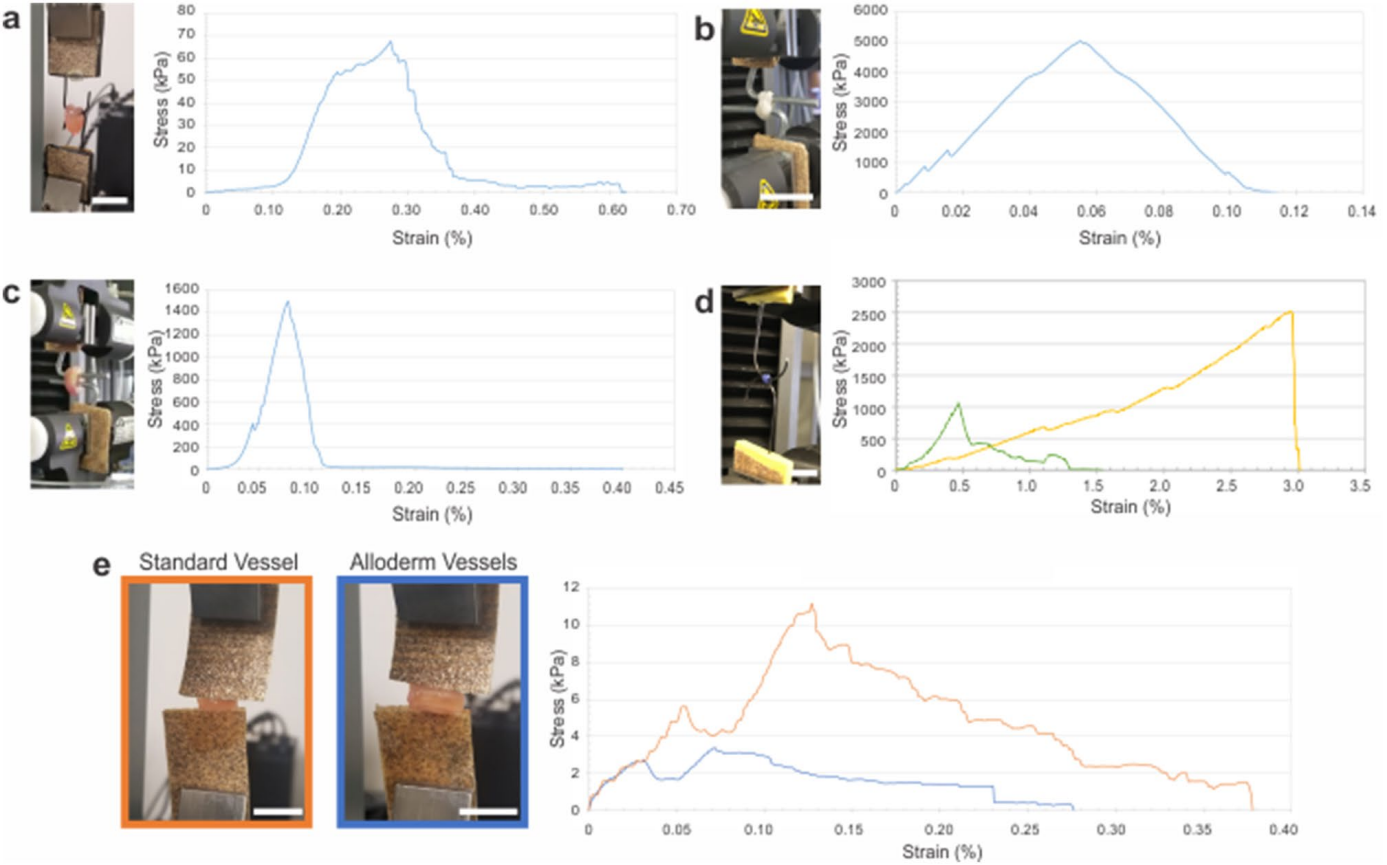
Significantly increased mechanical properties with inclusion of Alloderm into engineered vascular vessels. Average circumferential stress–strain curves for (**a**) standard vessels (n = 5), (**b**) Alloderm alone (n = 4), (**c**) Alloderm vessels (n = 5), and (**d**) human diabetic saphenous veins (green line; n = 6) and cadaver femoral arteries (yellow line; n = 8). (**e**) Average longitudinal stress–strain curves for standard and alloderm vessels; average circumferential stress–strain curves. Scale bars = 1 cm.

Engineered vessel tensile mechanics significantly improved with inclusion of Alloderm. Average stress–strain curves for rings without Alloderm, rings with Alloderm and Alloderm donuts are shown in Fig. 3a–e and summarized in Table 2. Interestingly, initial (failure strength 1; FS1) and complete (failure strength 2; FS2) failure points were noted for the vessel groups, indicating the point of failure of the first and last ring, respectively. These two failure points are of importance to note because the initial point of failure is vital information for clinical application, as is the catastrophic point of complete vessel failure. Average elastic modulus and ultimate tensile strength for vessels without Alloderm (n = 5) were 79.4 ± 11.6 kPa and 67.9 ± 9.78 kPa, respectively. The primary failure point and secondary failure point of standard rings were 49.8 ± 27.1 kPa and 3.22 ± 1.47 kPa, respectively. Average elastic modulus and ultimate tensile strength for Alloderm vessels (n = 5) were 3720 ± 687 kPa and 1500 ± 262 kPa, respectively. The primary failure point and secondary failure point for Alloderm vessels were 1400 ± 301 kPa and 4.77 ± 1.73 kPa, respectively (Supplemental Video 1). To compare the contribution of the Alloderm material alone in the vessels, 6 Alloderm donuts were adhered with Vetbond and tensile tested. Average elastic modulus and ultimate tensile strength of the 6 Alloderm donuts (n = 4) were 11.4 ± 1.96 MPa and 5050 ± 333 kPa, respectively. The primary failure point and secondary failure point were 4800 ± 384 kPa and 119 ± 39.2 kPa, respectively. Percent elongation for vessels without Alloderm, Alloderm vessels and 6 Alloderm donuts alone were 511 ± 64.9%, 286 ± 56.1% and 3730 ± 733%, respectively. To evaluate the Alloderm vessels compared to native vessels, human saphenous vein was also mechanically tested. Interestingly, the Alloderm vessels surpassed circumferential tensile mechanics of two native human vessels tested for comparison. Human saphenous vein (n = 6), which exhibited an elastic modulus of 2980 ± 409 kPa, ultimate tensile strength of 1060 ± 155 kPa and failure strength of 416 ± 157 kPa. The human saphenous vein had an average percent elongation of 821 ± 141%. Alloderm vessels exhibited a higher elastic modulus but lower ultimate tensile and failure strength in comparison to cadaver femoral artery (n = 8) which had an elastic modulus of 1280 ± 303 kPa, ultimate tensile strength of 2540 ± 748 kPa and failure strength of 2360 ± 773 kPa. The Alloderm vessels’ average percent elongation was similar to that of the human cadaver femoral artery (281 ± 36.9%).

**Table 2:** Average circumferential vessel material properties.

Group	E (kPa)	UTS (kPa)	FS Primary (kPa)	FS Secondary (kPa)	Percent Elongation (%)
Standard vessels (n = 5)	79.4 ± 11.6^a,b,c^	67.9 ± 9.78^a,b,c,d^	49.8 ± 27.1^a,b,d^	3.22 ± 1.47^b^	511 ± 64.9^a,b,c,d^
Alloderm vessels (n = 5)	3720 ± 687^a,e,g^	1500 ± 262^a,e,g^	1400 ± 301^a,e,f,g^	4.77 ± 1.73^e^	286 ± 56.1^a,e,f^
Alloderm alone (n = 4)	11,400 ± 1960^b,e,h,i^	5050 ± 333^b,e,h,i^	4800 ± 384^b,e,h,i^	119 ± 39.2^b,e^	3730 ± 733^b,e,i^
Human saphenous vein (n = 6)	2980 ± 409^c,h^	1060 ± 155^c,h,j^	416 ± 157^f^^,h,j^	N/A	821 ± 141^c,f,j^
Cadaver femoral artery (n = 8)	1280 ± 303^g^^,i,j^	2540 ± 748^d,g,i,j^	2360 ± 773^d,g,i,j^	N/A	281 ± 36.9^d, i, j^

^a^Statistically significant difference between Standard Vessels and Alloderm Vessels (E: p < 0.0001; UTS: p < 0.0001; FS Primary: p ≤ 0.01; FS Secondary: not significant; Percent Elongation: p < 0.0001).

^b^Statistically significant difference between Standard Vessels and Alloderm Alone (E: p < 0.0001; UTS: p < 0.0001; FS Primary: p < 0.0001; FS Secondary: p < 0.0001; Percent Elongation: p < 0.0001).

^c^Statistically significant difference between Standard Vessels and Diabetic Human Saphenous Vein (E: p < 0.0001; UTS: p < 0.01; FS Primary: not significant; Percent Elongation: p < 0.0001).

^d^Statistically significant difference between Standard Vessels and Cadaver Femoral Artery (E: not significant; UTS: p < 0.0001; FS Primary: p < 0.0001; Percent Elongation: p < 0.0001).

^e^Statistically significant difference between Alloderm Vessels and Alloderm Alone (E: p < 0.0001; UTS: p < 0.0001; FS Primary: p < 0.0001; FS Secondary: p < 0.0001; Percent Elongation: p < 0.0001).

^f^Statistically significant difference between Alloderm Vessels and Diabetic Human Saphenous Vein (E: not significant; UTS: not significant; FS Primary: p < 0.05; Percent Elongation: p < 0.0001).

^g^Statistically significant difference between Alloderm Vessels and Cadaver Femoral Artery (E: p < 0.001; UTS: p < 0.01; FS Primary: p ≤ 0.01; Percent Elongation: not significant).

^h^Statistically significant difference between Alloderm Alone and Diabetic Human Saphenous Vein (E: p < 0.0001; UTS: p < 0.0001; FS Primary: p < 0.0001; Percent Elongation: not significant).

^i^Statistically significant difference between Alloderm Alone and Cadaver Femoral Artery (E: p < 0.0001; UTS: p < 0.0001; FS Primary: p < 0.0001; Percent Elongation: p < 0.0001).

^j^Statistically significant difference between Diabetic Human Saphenous Vein and Cadaver Femoral Artery (E: p < 0.0001; UTS: p < 0.0001; FS Primary: p < 0.0001; Percent Elongation: p < 0.0001).

Longitudinal tensile mechanics representing strength along the length of the vessels was not significantly different in vessels without Alloderm compared to vessels with Alloderm (Fig. 3, Table 3). The longitudinal elastic modulus, ultimate tensile strength and failure strength for vessels without Alloderm (n = 5) were 26.1 ± 13.5 kPa, 11.2 ± 6.05 kPa and 1.54 ± 0.304 kPa, respectively. The longitudinal elastic modulus, ultimate tensile strength and failure strength for vessels with Alloderm (n = 5) were 10.7 ± 8.09 kPa, 3.47 ± 1.61 kPa and 1.41 ± 1.08 kPa, respectively.

**Table 3:** Longitudinal vessel material properties.

Group	E (kPa)	UTS (kPa)	FS (kPa)
Standard vessels (n = 5)	26.1 ± 13.5	11.2 ± 6.05^a^	1.54 ± 0.304
Alloderm vessels (n = 5)	10.7 ± 8.09	3.47 ± 1.61^a^	1.41 ± 1.08

^a^Statistically significant difference between Standard Vessels and Alloderm Vessels (E: no significance; UTS: p < 0.05; FS: not significant).

The force required to strain the engineered rings, engineered vessels and Alloderm alone to failure (Supplemental Fig. S1; Supplemental Tables S1 and S2) provide insight into the resultant mechanics and the effects of the difference in cross-sectional area on strength calculations. Significant differences in forces to obtain circumferential elasticity, tensile strength and failure strength were found between standard rings and Alloderm alone (p < 0.001). Standard rings had an average elastic force, ultimate tensile force and failure force of 0.127 ± 0.134 N, 0.273 ± 0.134 N and 0.162 ± 0.104 N, respectively. Alloderm alone had an average elastic force, ultimate tensile force and failure force of 21.9 ± 9.31 N, 12.7 ± 2.94 N and 11.8 ± 3.27 N, respectively. Although significant differences were seen in comparing material properties of Alloderm rings to Alloderm alone, when comparing associated forces no significant differences were found between ultimate tensile strengths (p = 0.102), and between Alloderm ring primary failure force and Alloderm alone failure force (p = 0.793). This indicates that the larger thickness of the Alloderm rings compared to the Alloderm donuts is responsible for the difference in calculation of strength due to the difference in cross-sectional area. Alloderm rings had an average elastic force, ultimate tensile force, primary failure force, and secondary failure force of 56.3 ± 6.58 N, 15.1 ± 0.677 N, 12.7 ± 1.94 N, and 0.0558 ± 0.0207 N, respectively. This indicates structural integrity of Alloderm was not compromised when in the rings, but rather the reduced mechanical properties can be attributed to increased cross-sectional area. Between standard rings and Alloderm rings, a significant difference was found between elastic force, ultimate tensile force and primary failure strength force (p < 0.001). These force outputs indicate the superior tensile mechanics from inclusion of Alloderm into the rings. Using an independent t-test with equal variances not assumed, a significant difference was found between standard rings failure strength and Alloderm ring secondary failure strength (p < 0.01), however, this can also be attributed to increased cross-sectional area. Using an independent t-test with equal variances not assumed, no significant difference was found between the standard ring’s failure force and Alloderm ring secondary failure force (p = 0.084). This indicates that the cells in the fibrin gel maintain their mechanical properties regardless of the inclusion of Alloderm. All together, these results indicate that Alloderm rings are a composite material comprised with material properties of strength from the Alloderm and ductility from the ring of cells organized in the fibrin gel.

### Rings and vessels histological analysis

Histological analysis of rings provided pertinent information on cellular and ECM protein content and organization in the engineered tissue. Vessel histology showed cellular and ECM organization across the multi-ring structure. Cross-sectional ring samples were stained with multiple stains. Hematoxylin and eosin was used to visualize overall cellular structure by staining nuclei deep purple and cytoplasm and extracellular matrix pink. Masson’s Trichrome and Picrosirius red stains were used to visualize ECM content and organization, by staining collagen blue and red, respectively. DAPI stains were used to clearly demarcate cell density and location.

**Figure 4:**
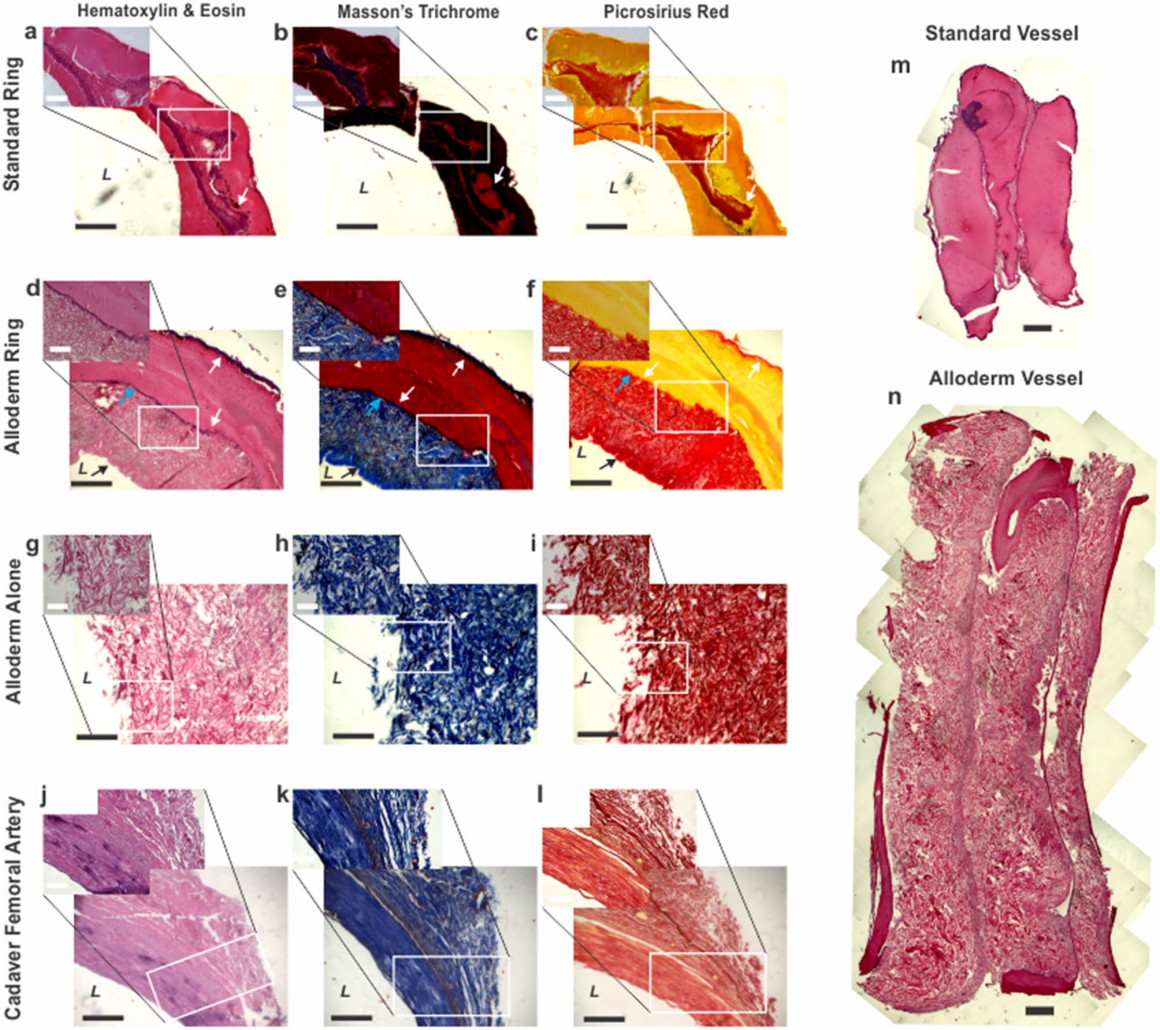
Extracellular matrix and cellular organization in engineered vascular rings and vessels improved with inclusion of Alloderm. H&E, trichrome and picrosirius red stains of (**a–c**) standard rings, (**d–f**) Alloderm rings, (**g–i**) Alloderm alone, and (**j–l**) cadaver femoral artery. Standard rings show well organized cells (white arrows) surrounded by fibrin gel (**a–c**). The addition of Alloderm (black arrows) further improved tissue organization by providing structural support for the cells (white arrows) and fibrin gel (blue arrows) around which to organize (**d–f**). Alloderm’s dense, organized collagen is evident in the stains of Alloderm alone (**g–i**). The Alloderm rings were the most similar in morphology and cellular organization to the cadaver femoral artery (**j–l**). H&E stained longitudinal sections of engineered vessels (**m,n**) show the ring-to-ring integration within the vessels, and the improvement of tissue organization with inclusion of Alloderm (**n**) compared to standard vessels without Alloderm (**m**). *L* indicates lumen side. White scale bars = 200 µm; black scale bars = 500 µm.

In the standard rings (Fig. 4a–c), fibroblasts self-organized into a band of cells surrounded by a layer of the fibrin gel. In rings with Alloderm (Fig. 4d–f), the organization of the ring from the lumen outward was first the Alloderm, followed by cells, then fibrin gel, and lastly more cells lining the outer diameter. Average ring thickness without and with Alloderm was 0.964 ± 0.170 mm and 2.35 ± 0.198 mm, respectively. Cell nuclei were seen located in the fibrin gel and Alloderm indicating cell migration. In rings with Alloderm, Trichrome stain showed a large blue band at the Alloderm, indicating its significant collagen content. In addition, a thin blue band was seen on the outer diameter suggesting collagen deposition by the cells in the Alloderm rings which was not evident in standard rings. This collagen deposition pattern was confirmed in picrosirius red stained sections where the red collagen-stained areas were seen co-localized with the cells and in parts of the hydrogel. In contrast, little positive collagen stain was seen in the standard rings without Alloderm. The same dense blue and red collagen network is seen in the Trichrome and Picrosirius red stains of Alloderm alone (Fig. 4h–i). Both trichrome and picrosirius red stains of the groups showed Alloderm alone had the highest percentage of area stained positive for collagen followed by Alloderm rings and then standard rings. Trichome stains of standard rings and Alloderm rings showed a significant difference (p < 0.001) in quantified collagen, 18.8 ± 4.77% and 65.6 ± 14.9%, respectively. Picrosirius red stains of rings without Alloderm and with Alloderm also showed significant differences (p < 0.001) in quantified collagen as 21.7 ± 8.27% and 66.7 ± 11.4%, respectively. Both ring groups additionally showed significant differences (p < 0.001) in percent area stained collagen from trichrome, 100 ± 0%, and picrosirius red, 100 ± 0%, in Alloderm alone. Quantified collagen from trichrome and picrosirius stains of human cadaver femoral artery showed a percentage of 73.9 ± 8.04% and 86.0 ± 6.98%, respectively, of collagen per area. Alloderm ring collagen percentage was not significantly different from the cadaver femoral artery, indicating similarity in collagen content. In contrast, standard rings and Alloderm alone did show significance difference in collagen content compared to the cadaver femoral artery (p < 0.001).

Longitudinal cross sections of vessels with and without Alloderm stained for H&E (Fig. 4m,n) showed successive rings composed of cells, fibrin gel, and Alloderm for the Alloderm rings. Areas of cellular density were evident by the purple nuclei stains. Standard vessels without Alloderm showed cellular organization as dense pockets of cells, whereas Alloderm vessels show more evenly distributed layers of cells.

**Figure 5:**
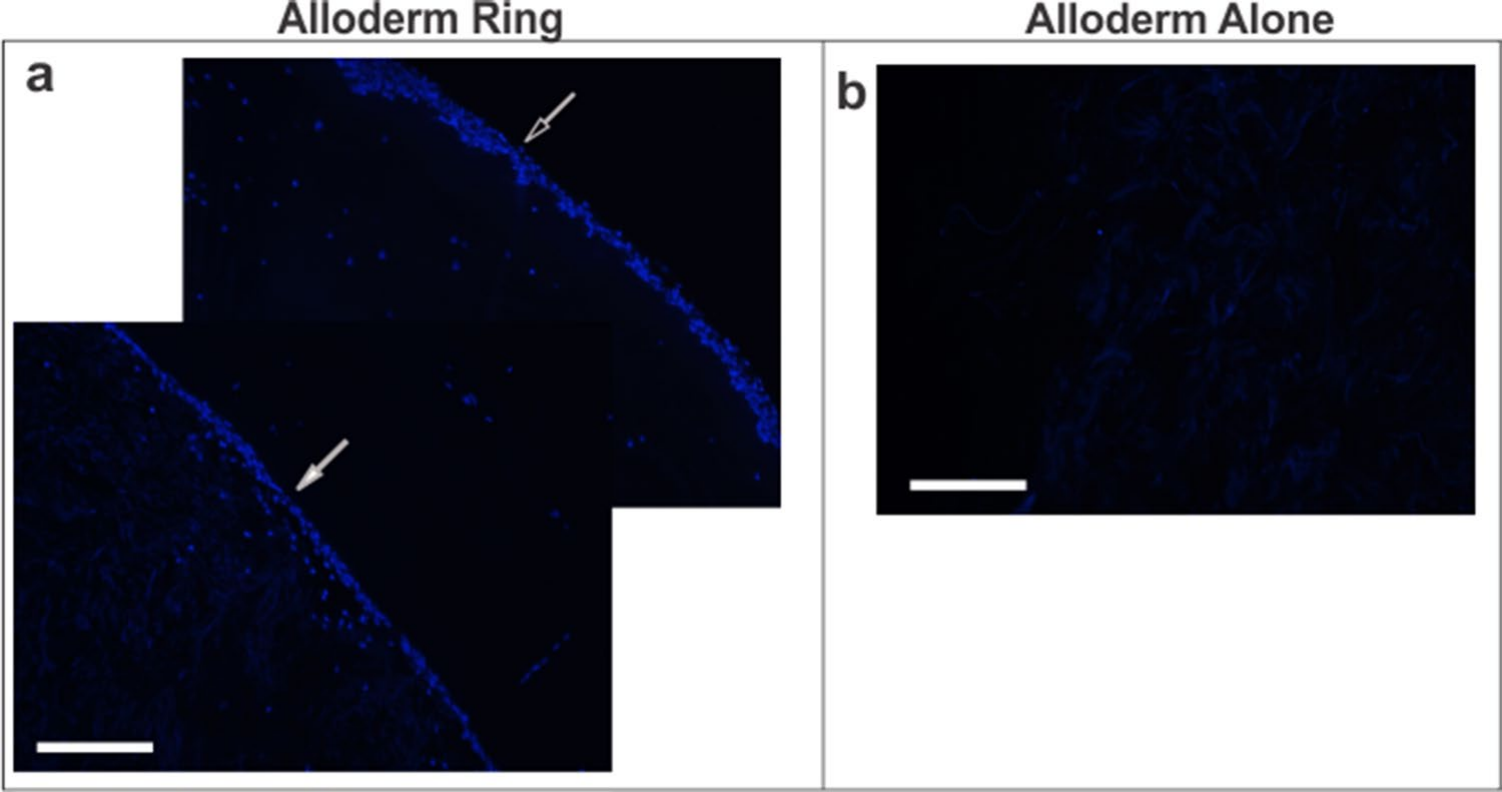
Cells infiltrated the Alloderm ECM in the engineered vascular tissue. DAPI stains of (**a**) an Alloderm-integrated vascular ring and (**b**) Alloderm alone. (**a**) Positive DAPI stain in Alloderm rings on the edge indicate cells seeded on the outer surface during the ring formation process (white open arrow), and positive DAPI stain in the Alloderm indicates cell infiltration (white closed arrow). (**b**) Lack of positive DAPI stain in the Alloderm alone verifies lack of cell presence prior to cell seeding, indicating that cells presence in the Alloderm in the rings (**a**) is due to cell infiltration. Scale bars = 200 µm.

DAPI fluorescent stains of cell nuclei in Alloderm rings showed the organization of the cells along the outer diameter of the rings (Fig. 5). In comparison, DAPI stains for Alloderm alone were negative indicating the absence of cells. Lack of positive stained DAPI in Alloderm alone indicates cell infiltration in Alloderm rings is from the ring making process rather than possible nuclear remnants from the original material of the decellularized Alloderm.

**Figure 6:**
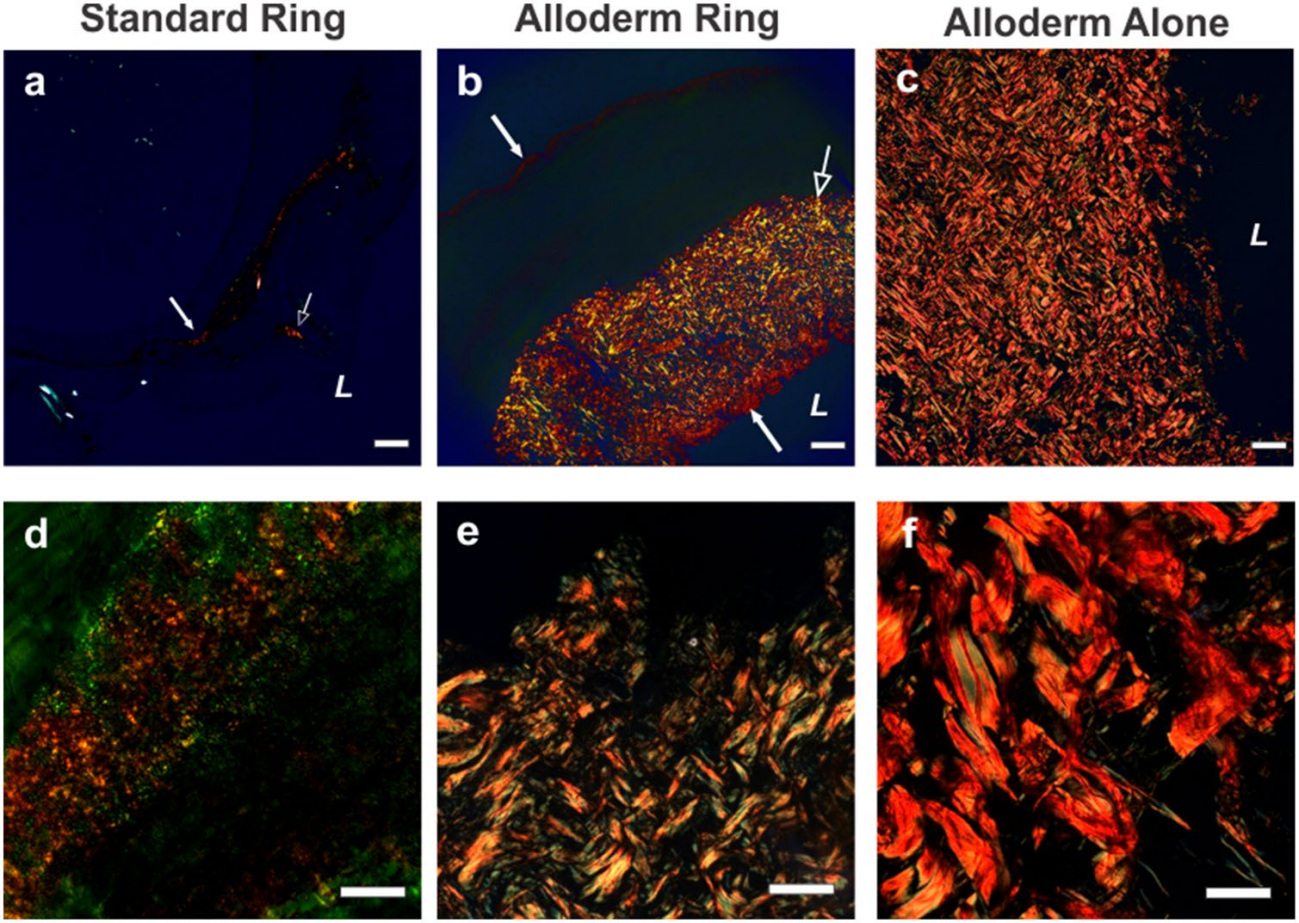
Increased collagen maturity of engineered vascular rings with incorporated Alloderm. Polarized light images of picrosirius red stained samples at (**a–c**) × 5 magnification and (**d–f**) × 40 magnification. Standard rings (**a,d**) exhibited mature red collagen fibers (white closed arrows) in the region of the cells and less mature yellow/orange fibers (white open arrows) in the fibrin gel. (**b,e**) Alloderm-integrated rings exhibited a mix of mature red collagen fibers and less mature yellow/orange fibers, along with green fibers indicating least maturity which was likely newly deposited by the cells. More mature red collagen fibers were present in the (**c,f**) Alloderm group, with very few green fibers. (**a–c**) scale bars = 200 µm; (**d–f**) scale bars = 100 µm; *L* indicates the lumen side.

Polarized light images of a Picrosirius red stained standard ring, Alloderm ring and Alloderm alone (Fig. 6) allowed for further assessment of collagen organization in the rings. Collagen fiber thickness can be visualized using polarized light microscopy of Picrosirius red stained tissues. More mature, thicker fibers appear orange to red whereas less mature, thinner fibers appear green to yellow. Rings without Alloderm primarily exhibited mature red collagen fibers in the region around the cells surrounded by areas of less mature yellow and orange collagen in the fibrin hydrogel. In the Alloderm alone, polarized light showed a dense red network of mature collagen. In the Alloderm ring, a dense red–orange network of collagen is seen in the Alloderm area, along with a lighter region of red fibers and orange fibers deposited by the cells surrounding the Alloderm. These results clearly show the enhanced collagen content provided by the inclusion of the Alloderm into the engineered tissue. Polarized light quantifications for percent area of fibers shows significant differences (p < 0.01) in red fibers for all three groups, in yellow fibers for all three groups, and in green fibers (p < 0.01) between standard rings and Alloderm alone and between standard rings and Alloderm rings. Standard rings contained 15.8 ± 7.85, 58.2 ± 13.9 and 19.0 ± 8.77 percent area of red, yellow and green collagen fibers, respectively, which was the highest yellow and green fiber content. Alloderm alone contained 91.3 ± 5.55, 7.49 ± 4.91 and 0.757 ± 0.791 percent area of red, yellow and green collagen fibers, respectively, which contained the highest red fiber composition. Alloderm rings contained 73.7 ± 5.49, 22.9 ± 5.41 and 3.47 ± 1.72 percent area of red, yellow and green collagen fibers, respectively, which contained higher red fiber content than similar green fiber content as Alloderm alone.

### Suture retention and burst pressure

**Figure 7:**
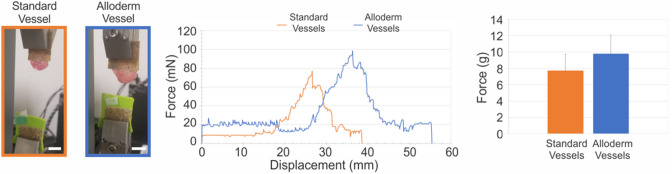
Average suture retention strength of engineered vessels increased with inclusion of Alloderm. Suture retention testing was performed on standard vessels (n = 5) and Alloderm vessels (n = 5). One end of the vessel was fixed to the tensile testing machine with sandpaper, with the suture glued to the other hook as shown. Both vessels experienced similar force output trends with respect to displacement. Alloderm vessels had a higher average maximum force compared to standard vessels. Scale bars = 1 cm.

**Figure 8:**
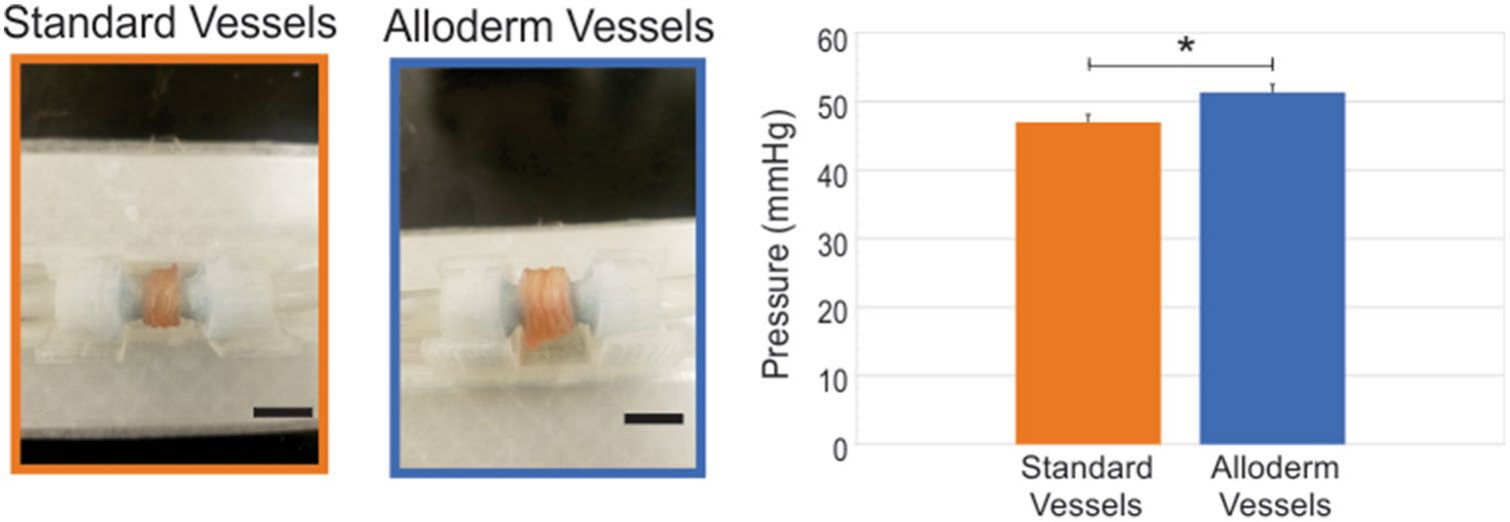
Burst pressure testing of engineered vessels. Standard vessels without (n = 5) and with Alloderm (n = 5) were placed under increasing pulsatile flow until failure. Vessels are shown loaded into the custom perfusion system used to perform the burst pressure tests. Alloderm vessels had significantly higher burst pressure compared to vessels without Alloderm (*p < 0.005). Scale bars = 1 cm.

No significant differences were found between vessels with and without Alloderm for suture retention (Fig. 7). Average suture retention for vessels without Alloderm (n = 3) was 7.73 ± 2.01 g-force. Average suture retention for vessels with Alloderm (n = 3) was 9.83 ± 2.25 g-force. In both vessel groups, suture retention failure points occurred in the area between the rings, at about 1 to 2 rings above the suture. However, there was a significant difference between burst pressure between vessels with and without Alloderm, with values of 51.3 ± 2.19 mmHg and 47.0 ± 1.14 mmHg, respectively (Fig. 8).

### Mechanical integrity maintained in Alloderm rings cultured long-term

**Figure 9:**
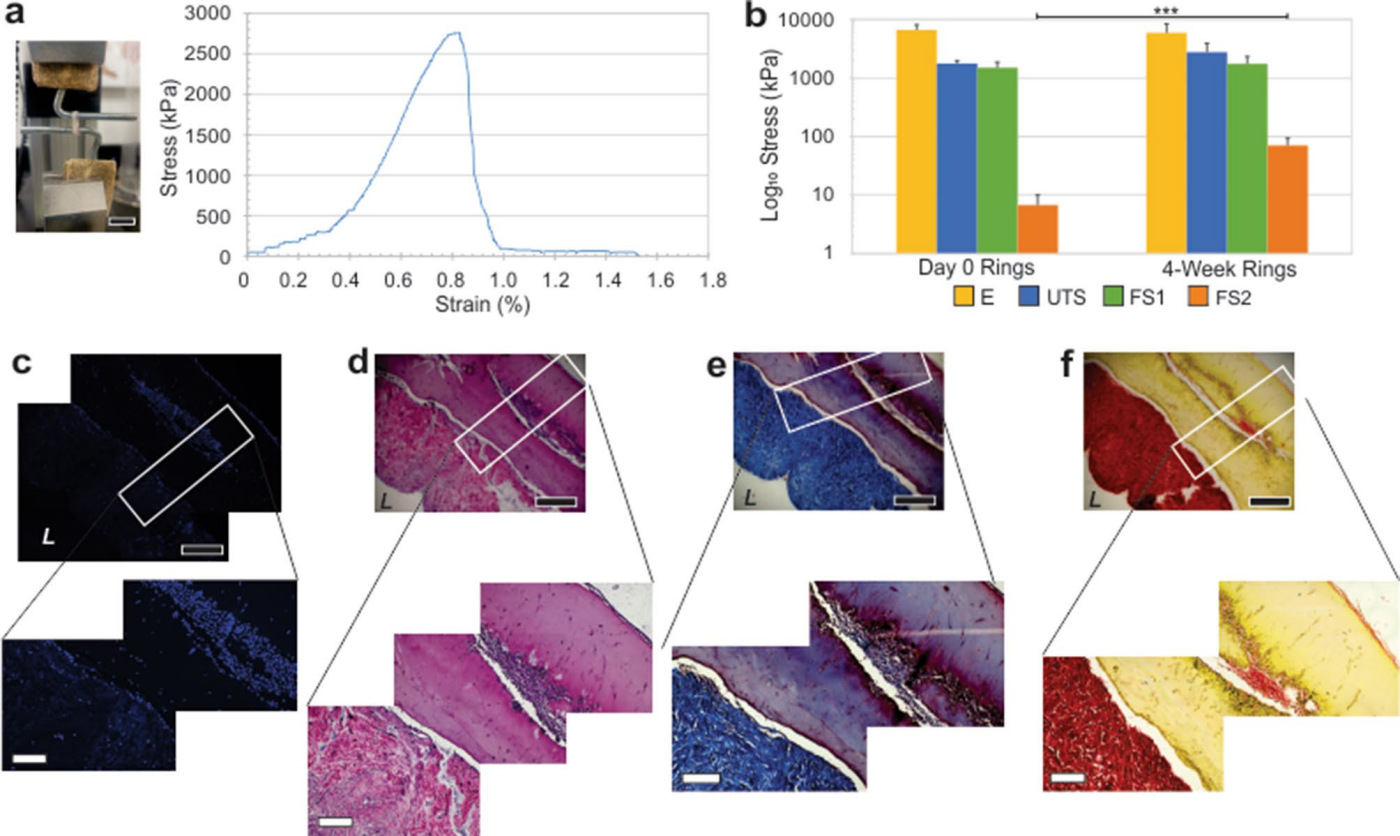
Engineered Alloderm rings exhibit long-term viability. Alloderm-integrated rings cultured for 4 weeks showed retainment of mechanical integrity. (**a**) A 4-week cultured Alloderm ring sample in the tensile setup with the associated average stress–strain curve. (**b**) Average elastic modulus (E), ultimate tensile strength (UTS), failure strength 1 (FS1), and failure strength 2 (FS2) for the day 0 and 4-week rings showing mechanics were maintained over the 4 week period; in fact, FS2 significantly increased after 4 weeks in culture indicating development of ECM strengthening the tissue. Histological analyses of 4-week rings stained for (**c**) fluorescence DAPI, (**d**) H&E, (**e**) Masson’s Trichrome, and (**f**) picrosirius red showing maintenance of tissue integrity and cellularity over time. Black scale bars = 500 µm; white scale bars = 200 µm.

To test long-term viability, Alloderm rings were cultured for 4 weeks and mechanically tested (Fig. 9). Alloderm rings cultured for 4 weeks demonstrated similar mechanical properties to Alloderm rings tested immediately following ring formation (at day 0) as determined by an independent sample t-test (p > 0.5). The long-term cultured rings exhibited an elastic modulus, ultimate tensile strength, primary failure strength, and percent elongation of 5910 ± 2560 kPa, 2760 ± 1130 kPa, 1750 ± 588 kPa, and 170 ± 53.2%, respectively. The secondary failure strength, a material property associated with the fibrin gel component, was significantly higher in the 4-week cultured rings compared to day 0 rings, exhibiting a FS2 of 70.5 ± 24.6 (p < 0.001).

### Physiologically relevant long vessel

**Figure 10:**
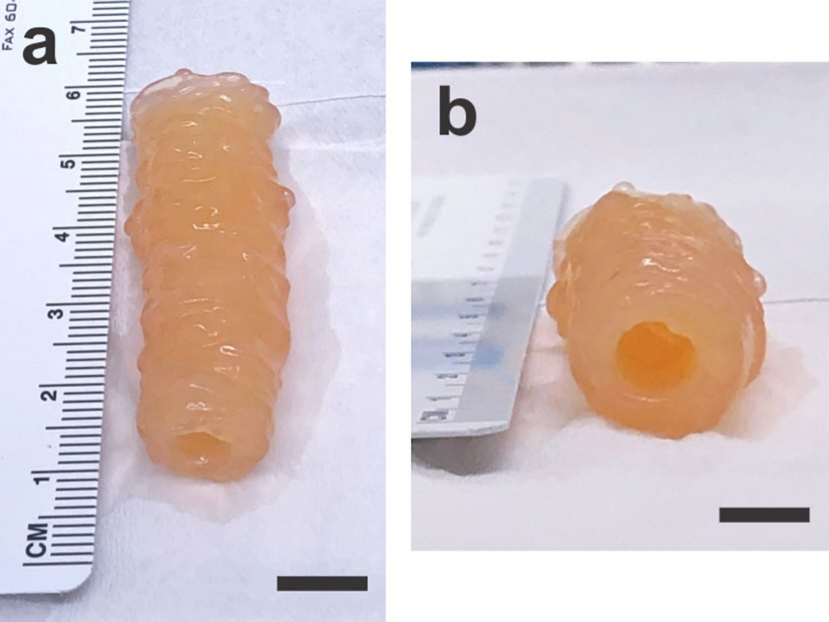
Physiologically relevant length engineered vessel. A 5 cm long adventitia vessel was created, demonstrating the capability of the Ring Stacking Method to create engineered vessels in lengths suitable for human implantation. (**a**) Longitudinal view; scale bar = 1 cm. (**b**) Luminal view; scale bar = 5 mm.

A 5 cm long vessel was fabricated (Fig. 10) to demonstrate the capability of the Ring Stacking Method to generate vessels in lengths suitable for human implantation. Depending on the implantation site, degree of injury and vessel to be repaired, varying graft lengths are needed, most typically ranging from 2 to 10 cm^32^. Hence, the 5 cm long vessel built shows clinical feasibility of our adventitia vessels.

### Endothelialization of adventitia vessels

**Figure 11:**
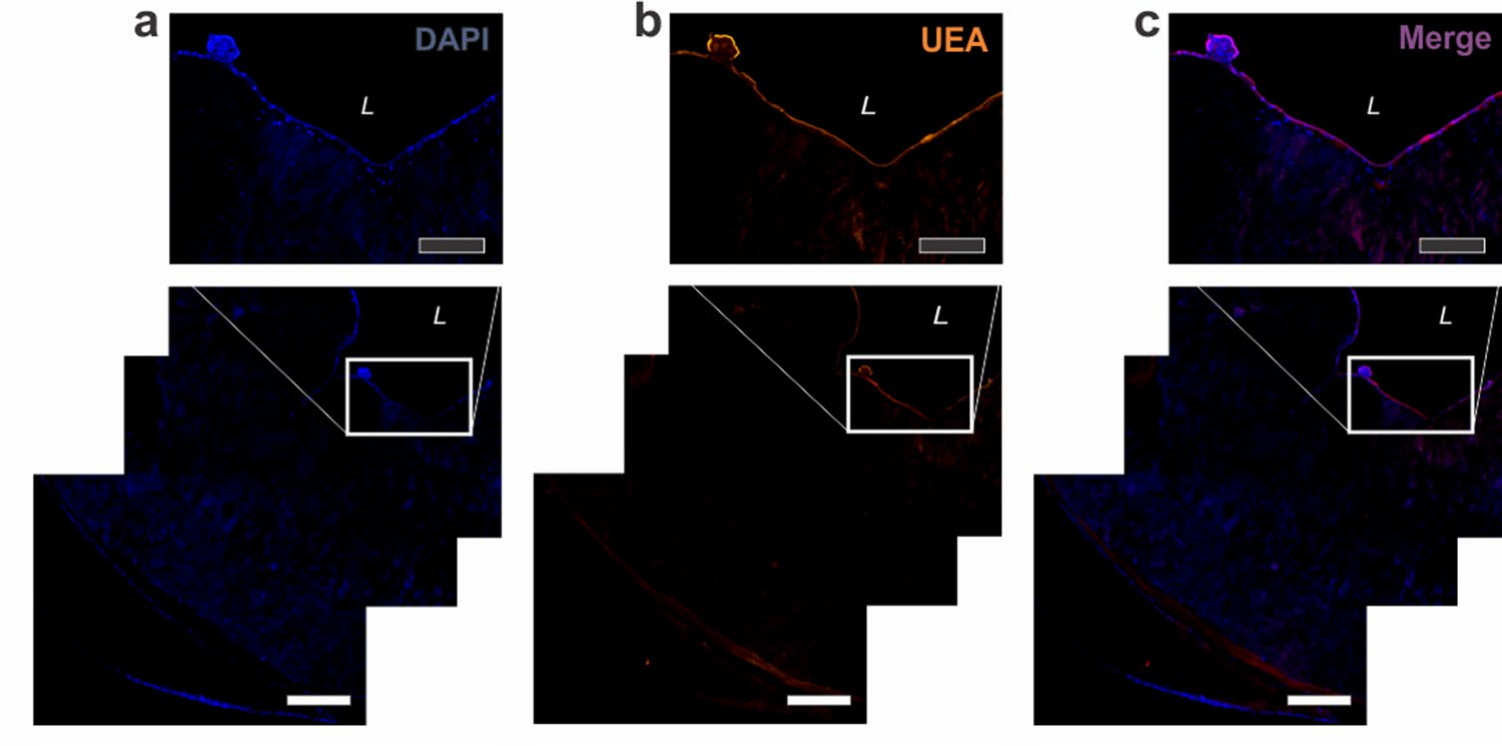
Endothelialization of Alloderm-integrated vessels. Vessel lumen was successfully endothelialized with human umbilical cord endothelial cells (HUVECs). UEA-1 stain showed complete coverage of the vessel lumen with HUVECs. Black scale bars = 500 µm; white scale bars = 200 µm.

Human umbilical vein endothelial cells were successfully seeded into Alloderm vessels (Fig. 11). Formation of a single endothelial cell layer was demonstrated in the lumen of the vessel, evidenced by positive staining for UEA-1 throughout the vessel lumen. This indicates that in vivo the Alloderm vessels have the potential to successfully endothelialize.

### Platelet adhesion assay

**Figure 12:**
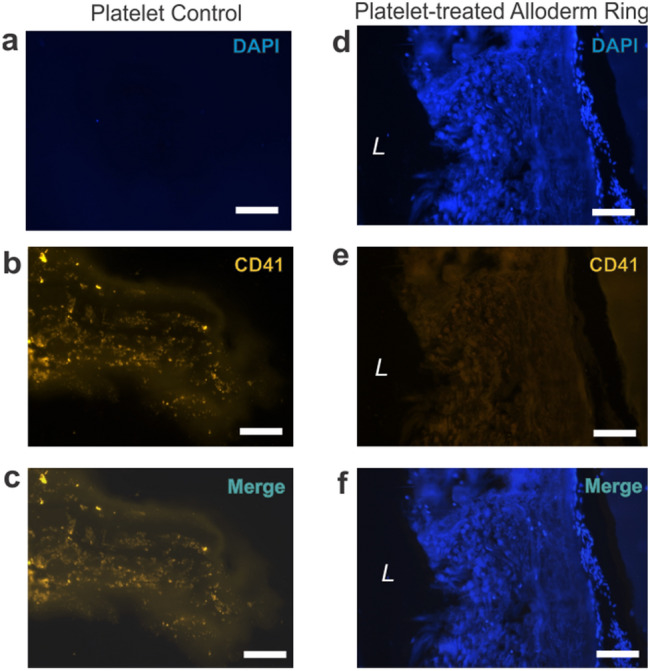
Alloderm rings did not exhibit adhesion to human platelets. Compared to the platelet control (**a–c**), Alloderm rings cultured in human platelets for 24 h (**d–f**) did not exhibit platelet adhesion as evidenced by negative CD41 anti-platelet stain (**e**). DAPI stain demarcated nuclei only evident in the Alloderm rings (**d**). CD41 stain was only positive in the platelet control (**b**). Scale bars = 200 µm.

To test thrombogenicity, Alloderm rings were submerged in a fresh human platelet concentrate for 24 h. Negative CD41 anti-platelet antibody stains of the Alloderm rings indicated no platelet adherence (Fig. 12). The control of platelets embedded in a fibrin hydrogel stained positive for CD41, validating efficacy of the antibody. These results suggest that when implanted in vivo, Alloderm vessels would not inherently cause thrombus formation.

## Discussion

The Alloderm ECM resulted in the ideal material for adding strength to tissue engineered blood vessels. Given the Alloderm’s pliability and strength, it easily integrated into the aggregating ring structures while concurrently offering sufficient support and strength for the final tissue. The Alloderm ECM organized into the lumen side of the ring and was lined by the fibrin gel and cells on the outer edge. This organization allowed the Alloderm to serve as the main component to counteract forces exerted on the tissue while the cells on the outer edge provided elasticity. This observation was supported by the tensile results showing two main failure points- first, of the stiffer Alloderm material and then, of the more elastic cellular component. Interestingly, vessels with the Alloderm material showed increased collagen content and encouraged collagen deposition by the cells as seen in the additional collagen lining the cells. Additionally, cell nuclei were seen located in the fibrin gel and Alloderm, which indicates that the extracellular matrix encouraged cell migration and infiltration.

Inclusion of the Alloderm into the engineered rings significantly improved circumferential tensile mechanics compared to standard rings. This is due to the significant increase in the load-bearing collagen extracellular matrix protein provided by the Alloderm. These results are quite encouraging to progress towards clinical application, since the incorporation of the Alloderm elevated the engineered vessels’ tensile strength to 1500 ± 334 kPa, which allows the engineered vessel to meet the comparative native adventitia tensile strength of 1430 ± 604 kPa^12^. The other important parameter for clinical application is the burst pressure. There was a significant difference in burst pressure with the addition of the Alloderm—however, the burst pressure needs further improvement in order to meet human blood pressure values. Our lab is currently testing methods to improve burst pressure in our vessels with promising preliminary results, which is the focus of an upcoming follow-up study. Nevertheless, the current burst pressure strength of 51.3 ± 2.19 mmHg of the Alloderm vessels is encouraging as it approaches human diastolic blood pressure values.

Interestingly, Alloderm rings exhibited a lower ultimate tensile strength and failure strength than Alloderm alone. This is due to the differences in the cross-sectional area of each group. Stress is calculated as the force divided by the cross-sectional area, meaning that larger cross-sectional areas result in lower apparent stress. The rings have an inherent larger cross-sectional area due to the additional thickness of the cells and hydrogel compared to the Alloderm alone. Thus, the thicker the tissue, the larger the cross-sectional area and thus the smaller the calculated stress for a given force applied. This effect directly correlates to the parameter of strength which is determined from the stress–strain curve, as stress is a normalized factor, however, in cases such as this, force needed to cause tissue failure also provides useful information.

In the longitudinal direction, no significant differences were observed in forces associated with the elastic modulus and ultimate tensile strength between standard vessels and Alloderm rings, meaning that the addition of the Alloderm did not affect longitudinal vessel mechanics. Longitudinal strength of the engineered vessels with or with Alloderm could be improved. However, in vivo the vessels would not be subjected to major forces in that direction. Also, it has been shown that engineered vessels after implantation are remodeled with endogenous cell engraftment which further strengthens the tissue in all directions^2^. Regardless, the current study in our lab to improve burst pressure will concurrently strengthen the vessels in the longitudinal direction. Suture retention was not significantly different between standard engineered vessels and Alloderm vessels likely because suture retention relies on longitudinal structural components, similar to burst pressure strength. Hence, since burst pressure was not significantly different, it is not surprising that suture retention strength followed a similar trend.

The 4-week long cultured Alloderm rings exhibited retainment of mechanical properties over time. Failure strength 2 correlates with the strength of the fibrin gel in the rings. The increase in failure strength 2 in Alloderm rings indicates increased ECM deposition around the rings by the cells. The biological environment promotes tissue growth and ECM deposition, hence the engineered vessels will be further strengthened upon implantation in the future. Rings were chosen in this test because any potential mechanical changes over time would not exhibit a difference in rings compared to vessels.

The 5 cm long vessel built is capable of accommodating common repair of vessels such as the popliteal artery, femoral artery and iliac artery. In practice, any length vessel can be built using our methods by simply adding additional rings to the vessel, thus allowing for our methods to accommodate any vessel repair length needed. Moreover, vessel lumen can easily be modified by changing dimensions of the posts the vascular rings are fabricated around, furthering applicability of our vessels to accommodate repair of vessels of differing lumen sizes from small-diameter to larger diameter grafts, as was demonstrated in our previous work of fabricating vessels of lumen size 6, 10 and 20 mm^29^.

Successful endothelialization of the vessels show that the vessels readily support formation of an intima, which is pertinent for establishing proper hemodynamics. Endothelialization of grafts has shown to aid in increased patency rates^33^. Thus, demonstration of successful endothelialization of our Alloderm-integrated adventitia vessels provides further evidence of clinical applicability. The negative platelet adhesion test results further support patency of our engineered vessels, even with the inclusion of the Alloderm material. As one of the primary steps in coagulation, which can lead to vessel occlusion, the inherent characteristic of the adventitia vessels to not preferentially adhere platelets is advantageous.

In regard to in vivo performance, Alloderm has extensively previously been studied for its effects in vivo and in clinical application in human patients^34–38^. Alloderm has been used to in the clinic since 1994 and continues to be used today. The primary findings from the in vivo studies and clinical data are that Alloderm promotes neovascularization and fibroblast infiltration; and reduces inflammation and fibrosis compared to controls. Clinically, Alloderm has shown to integrate well into host tissue and has not, in some studies, shown any safety concerns in a 5-year follow-up. These positive attributes of Alloderm in vivo show promise for our Alloderm-integrated vessels to perform advantageously once implanted. Animal studies are planned in the near future to fully test our Alloderm vessels and determine outcomes following implantation.

## Conclusions

Here, we demonstrate the incorporation of commercially available decellularized extracellular matrix Alloderm to significantly improve the mechanical properties of our engineered biological vascular grafts, a pre-requisite for meaningful clinical utility. Additionally, the added ECM advantageously increased collagen content, improved ECM organization, increased mature collagen content, encouraged cell engraftment, and demonstrated the capability of endothelialization. Our ongoing work on methods to improve burst pressure strength currently shows promising preliminary results, further increasing its relevance for prospective patient applications.

## Supplementary Information

Supplementary Tables.

Supplementary Figure.

Supplementary Video 1.
