# Supplementary material for: Decellularized dermis extracellular matrix alloderm mechanically strengthens biological engineered tunica adventitia-based blood vessels
Source: Sci Rep. 2021 May 31;11:11384. doi: 10.1038/s41598-021-91005-9 (PMC8166942; doi:10.1038/s41598-021-91005-9)
Supplement: Supplementary file 1 — Supplementary Tables. [file 41598_2021_91005_MOESM1_ESM.pdf]

Decellularized Dermis Extracellular Matrix Alloderm as a Scaffold for Biological Engineered Blood Vessels

Bijal Patel[1], Bryan T. Wonski[1], Dan M. Saliganan[1], Ali Rteil[2], Loay S. Kabbani[2], Mai T. Lam[1,*]

[1]Department of Biomedical Engineering, Wayne State University, Detroit, MI
[2]Department of Vascular Surgery, Henry Ford Health System, Detroit, MI

Addresses:

Bijal Patel, M.S.E.
Wayne State University
818 W. Hancock Street
Detroit, MI 48201 USA
bijal.patel3@wayne.edu

Bryan T. Wonski, M.S.E.
Wayne State University
818 W. Hancock Street
Detroit, MI 48201 USA
wonski1bt@wayne.edu

Dan M. Saliganan, M.S.E.
Wayne State University
818 W. Hancock Street
Detroit, MI 48201 USA
saliganan1101@gmail.com

Ali Rteil, M.D.
2799 W Grand Blvd
Detroit, MI 48202 USA
ali.rteil@gmail.com

Loay Kabbani, M.D.
2799 W Grand Blvd
Detroit, MI 48202 USA
LKABBAN1@hfhs.org

Corresponding author:
Mai T. Lam, Ph.D.
Wayne State University

818 W. Hancock Street
Detroit, MI 48201 USA
mtlam@wayne.edu
Phone: (313) 577-0118
Fax: (313) 577-8333

Supplemental Table 1: Circumferential Ring Forces of Material Properties

Group	E Force (N)	UTS Force (N)	FS Primary force (N)	FS Secondary Force (N)
Standard Rings (n=5)	0.127 ± 0.0419[*‡]	0.273 ± 0.134[*‡]	0.162 ± 0.104[*‡]	N/A
Alloderm Rings (n=5)	56.3 ± 6.58[*†]	15.1 ± 0.678[*]	12.7 ± 1.94[*]	0.0559 ± 0.0207
Alloderm Alone (n=4)	21.9 ± 9.31[†‡]	12.7 ± 2.94[‡]	11.8 ± 3.27[‡]	N/A

*Statistically significant difference between Standard rings and Alloderm rings (E Force: $p<0.0001$; UTS force: $p<0.0001$; FS Primary force: $p<0.0001$)

† Statistically significant difference between Alloderm rings and Alloderm alone (E Force: $p<0.0001$, UTS force: not significant, FS Primary force: not significant)

‡ Statistically significant difference between Standard rings and Alloderm alone (E Force: $p\leq0.001$, UTS force: $p<0.0001$, FS Primary force: $p<0.0001$)

Supplemental Table 2: Longitudinal Vessel Forces of Material Properties

Group	E Force (N)	UTS Force (N)	FS Force (N)
Standard Vessels (n=5)	0.544 ± 0.282	0.234 ± 0.126	0.0322 ± 0.00636
Alloderm Vessels (n=5)	0.813 ± 0.616	0.258 ± 0.111	0.107 ± 0.0824

No statistically significant difference between Standard Vessels and Alloderm Vessels (E Force; UTS force; FS force)
