# Supplementary material for: Decellularized dermis extracellular matrix alloderm mechanically strengthens biological engineered tunica adventitia-based blood vessels
Source: Sci Rep. 2021 May 31;11:11384. doi: 10.1038/s41598-021-91005-9 (PMC8166942; doi:10.1038/s41598-021-91005-9)
Supplement: Supplementary file 2 — Supplementary Figure. [file 41598_2021_91005_MOESM2_ESM.pdf]

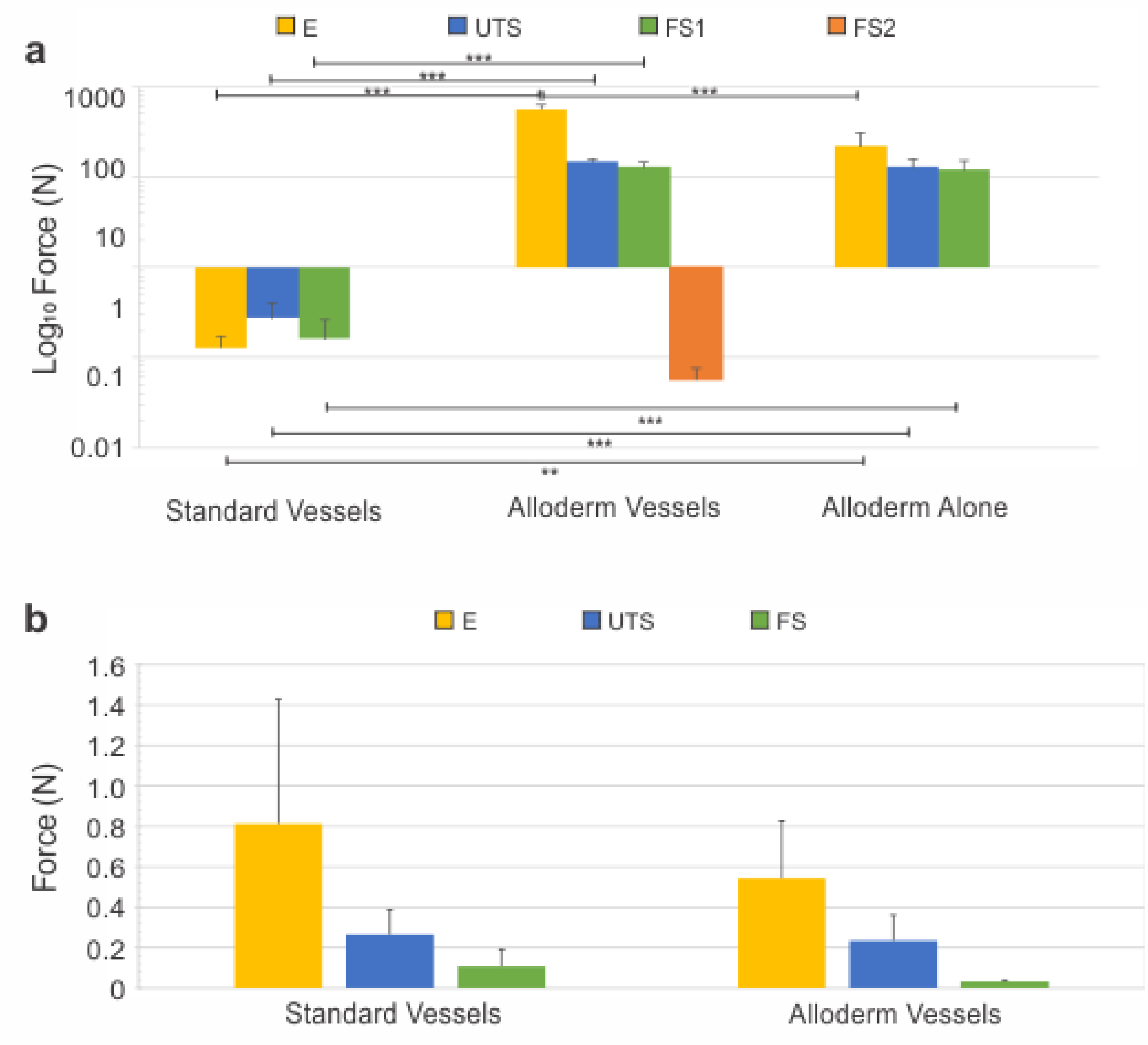

Supplemental Figure 1. Circumferential ring and longitudinal vessel tensile forces. Average circumferential ring (**a**) and longitudinal vessel (**b**) forces associated with tensile material properties. Significant differences were found for all material properties' forces between standard rings and Alloderm rings, and between standard rings and Alloderm alone. No significant difference in forces were found for Alloderm rings and Alloderm alone for the ultimate tensile strength and failure strength. Failure strength of standard rings and secondary failure strength of Alloderm rings were similar. There were no significant differences in the force output of the longitudinal material properties between vessels without and with Alloderm (**b**). *p < 0.01; **p < 0.001; ***p< 0.0001.
